# Altered somatosensory evoked potentials associated with improved reaction time in a simple sensorimotor response task following repetitive practice

**DOI:** 10.1002/brb3.1624

**Published:** 2020-06-25

**Authors:** Mayu Akaiwa, Koki Iwata, Hidekazu Saito, Takeshi Sasaki, Kazuhiro Sugawara

**Affiliations:** ^1^ Graduate School of Health Sciences Sapporo Medical University Sapporo Japan; ^2^ Department of Occupational Therapy School of Health Science Sapporo Medical University Sapporo Japan; ^3^ Department of Physical Therapy School of Health Science Sapporo Medical University Sapporo Japan

**Keywords:** N140, N250, P300, reaction time task, somatosensory evoked potentials

## Abstract

**Introduction:**

Repetitive practice of sensorimotor tasks is widely used for neurorehabilitation; however, it is unknown how practice alters sensory processing (e.g., recognition, discrimination, and attentional allocation) and associated cognitive processing, such as decision‐making. The purpose of this study was to investigate whether long‐latency somatosensory evoked potentials (SEPs) reflecting sensory processing, attention, and decision‐making are altered by sensorimotor learning.

**Methods:**

Fifteen participants performed a simple sensorimotor response task (thumb opposition in response to surface electrical stimulation), with experimental recording sessions before and after three days of practice. We then compared multiple SEP waveforms and reaction times (RTs) between pre‐ and postpractice trials.

**Results:**

The RT was reduced after practice of three days, and we found a significant positive correlation between ΔRT and ΔN140_lat_ at F3, Cz, and C3′, ΔRT and ΔN250_lat_ at F3, and there was a significant negative correlation between ΔRT and ΔP300_amp_ at C3′.

**Conclusion:**

The present study suggests that motor learning improves somatosensory processing and attentional allocation via neuroplasticity and that these alterations are reflected by specific SEP changes.

## Graphical abstract

This article shows that repetitive practice of sensorimotor task practice for 3 days changes reaction times and long latency somatosensory evoked potential components. Motor learning can be altered task‐relevant sensorimotor networks.

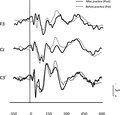

## 1 INTRODUCTION

Improved sensorimotor performance is a major aim of rehabilitation; however, quantitative tests for evaluating gains are lacking. Reaction time (RT) is widely used as a general measure of sensorimotor performance. It is commonly defined as the time elapsed from stimulus presentation to the onset of the electromyogram (EMG) response (i.e., the earliest sign of muscle activity). The relationship between RT and somatosensory processing efficiency has been examined in several studies using somatosensory evoked potentials (SEPs) and somatosensory evoked fields (SEFs). Hiraoka, Kori, and Sato (2017) investigated SEPs during rest and voluntary movement and reported that the amplitudes of N18 and P40 were significantly larger during short‐RT (fast motor response) trials compared to longer‐RT (slow motor response) trials. Similarly, Kida et al. (2003) and Kida, Nishihara, Hatta, and Wasaka (2003) recorded the event‐related potentials (ERPs) during a somatosensory discrimination task and reported that P300 was larger in fast‐RT trials than in slow‐RT trials. Sugawara et al. (2013) found that both RT and activation latency of the posterior parietal cortex (PPC), an area related to visuomotor integration and control, were significantly reduced after three days of visuomotor task repetition. Collectively, these results demonstrate that sensorimotor task practice induces neuroplastic changes that improve sensory processing. Yamashiro et al. (2013) compared baseball players to athletes in sports such as track and field events and soccer who do not require quick responses to hand sensation using a simple hand RT task, and they found shorter RT and reduced P100 and N140 peak latencies in the baseball group. It has been posited in previous studies that changes in P100 and N140 following somatosensory stimulation are related to a passive shift in attention (Kekoni, Hamalainen, McCloud, Reinekainen, & Naatanen, 1996) and that the amplitude of N140 in particular increases with spatial selective attention (Michie, 1984). In addition, N250 and P300 reflect active target detection by attentive processing, and P300 is associated with working memory (Donchin & Coles, 1988; Kekoni et al., 1996). It has been reported in previous studies that decreasing the RT through training also reduces ERP latencies in athletes, but no study has investigated SEP changes in nonathletes after brief training. We hypothesized that motor practice and a concomitantly shorter RT would increase the amplitude and reduce the latency of various SEP waveforms reflecting stimulus processing, attention, and/or response decisions.

## 2 METHODS

### 2.1 Participants

The study participants were 15 healthy young adults (age [mean ± standard deviation]: 22.5 ± 3.1 years; all right‐handed; 10 males, 5 females), all of whom provided written informed consent. The study conformed to the Declaration of Helsinki and the Code of Ethics of the World Medical Association and was approved by the ethics committee of Sapporo Medical University (No. 29‐2‐58).

### 2.2 Experimental procedure

The participants sat on a reclining chair and were instructed to relax during the SEP measurements. Then, they performed a simple sensorimotor response task, in which they performed right thumb palmar abduction as quickly as possible in response to the electrical stimuli delivered to the right median nerve at the right wrist. The duration of the electrical stimulus was 0.2 ms, and the intensity of the stimulus was set so that the amplitude of the evoked M‐wave in the abductor pollicis brevis muscle was 1 mV (Hiraoka et al., 2017). The interstimulus interval (ISI) was varied randomly between 300 and 1,000 ms to prevent habituation of the task. Between each set of 60 trials, an intermission was allowed to minimize the effect of fatigue. In total, six sets were conducted during the prepractice (Pre) period, which is control, and the average SEP amplitudes, SEP latencies, and RTs were recorded. The subjects then practiced this task for three days at our laboratory which recorded RTs. We then recorded the same metrics (SEPs and RTs) again after practice (Post). Between the pre‐  and postpoints, we recorded during a continuous period or every other day.

### 2.3 Recordings and analysis

All electrophysiological recordings were acquired using the Neuropack system (Nihon Kohden, Tokyo, Japan). The electroencephalography (EEG) was measured by Ag/AgCl electrodes placed over three scalp sites—F3, Cz, and C3′ (2 cm posterior to C3)—according to the International 10–20 system, and another electrode was placed on the left earlobe (A1) as a reference. The electrode impedance at all recording sites was maintained below 5 kΩ. The electrooculogram (EOG) was recorded from the right suborbital region. Trials in which the EOG waveform exceeded 50 µV were rejected. EEG signals were recorded with a 2–2,000 Hz band‐pass filter at a sampling rate of 10 kHz and analyzed with low‐pass filtering at 100 Hz. The analysis period of SEPs was from 100 ms before to 600 ms after the stimulus onset. The 100 ms period before the stimulus onset was used as the baseline. The amplitudes of SEPs were measured from baseline to peak. The average of more than 300 recordings was obtained during pre‐ and postpractice sessions for all subjects. The time windows (relative to the stimulus onset) for measuring peak amplitudes and latencies were as follows: (Kida et al., 2003; Kida et al., 2003; Yamashiro et al., 2013): P100 (50–120 ms), N140 (100–190 ms), N250 (180–280 ms), and P300 (250–500 ms). We defined the changes (Δ) in all metrics as postpractice–pre‐practice. The EMG was recorded from the right abductor pollicis brevis muscle. We calculated the time from the median nerve stimulus onset to the EMG onset as the RT. Trials with RTs shorter than 50 ms and longer than 400 ms were excluded from the analysis (Yotani et al., 2011). The EMG signals were recorded with a 1–300 Hz band‐pass filter at a sampling rate of 1,000 Hz. The mean RT and SEP waveforms were compared within subjects by paired *t* tests. Moreover, to test the normality of distribution, the Shapiro–Wilk test was used for the change in RT and the changes in P100, N140, N250, and P300 amplitudes and their latencies. If the distribution was normal according to the Shapiro–Wilk test, Pearson's correlation test was performed to evaluate the correlations between the change in RT and the changes in P100, N140, N250, and P300 latencies and their amplitudes. If the distribution was not normal, Spearman's correlation test was performed.

## 3 RESULTS

**FIGURE 1:**
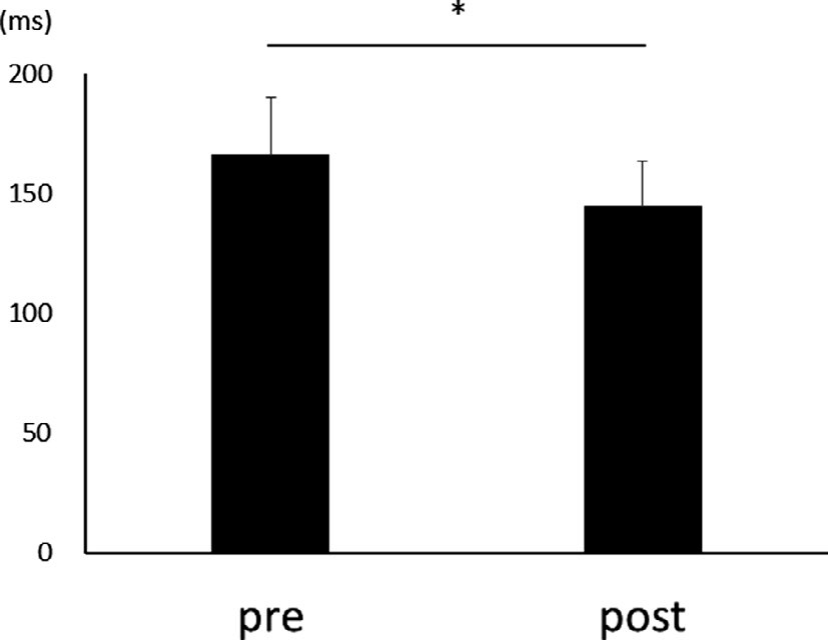
Mean reaction times (RTs) before (Pre) and after (Post) three days of practice (* *p* = .0003)

**FIGURE 2:**
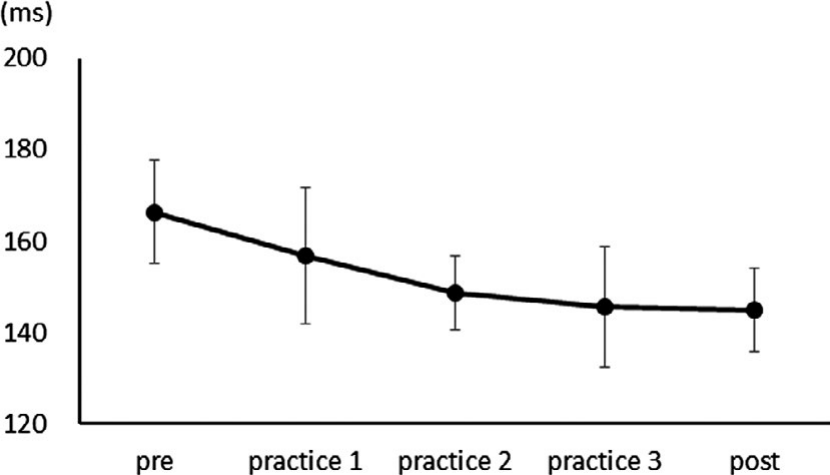
Averaged learning curve of RT in all participants

**FIGURE 3:**
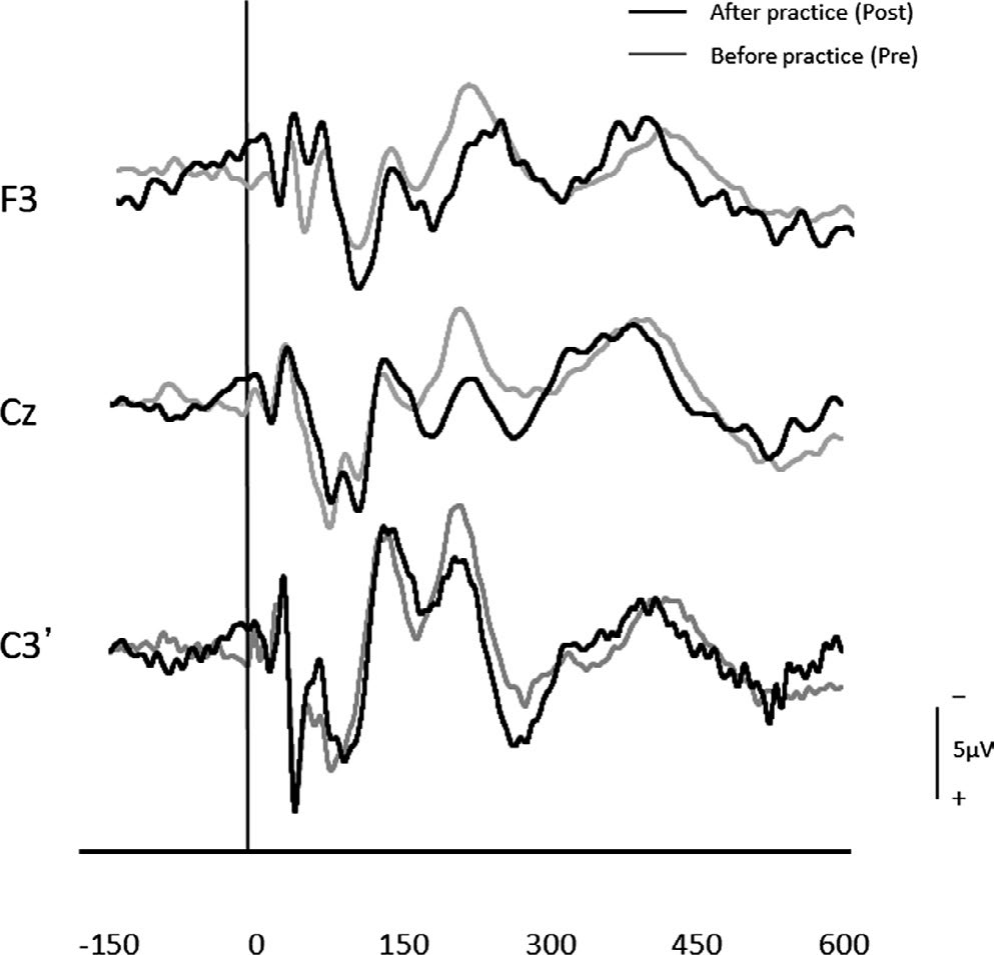
Typical examples of somatosensory evoked potentials (SEPs) in a sensorimotor response task. The gray and black lines represent SEPs recorded before practice (Pre) and after three days of practice (Post), respectively

**FIGURE 4:**
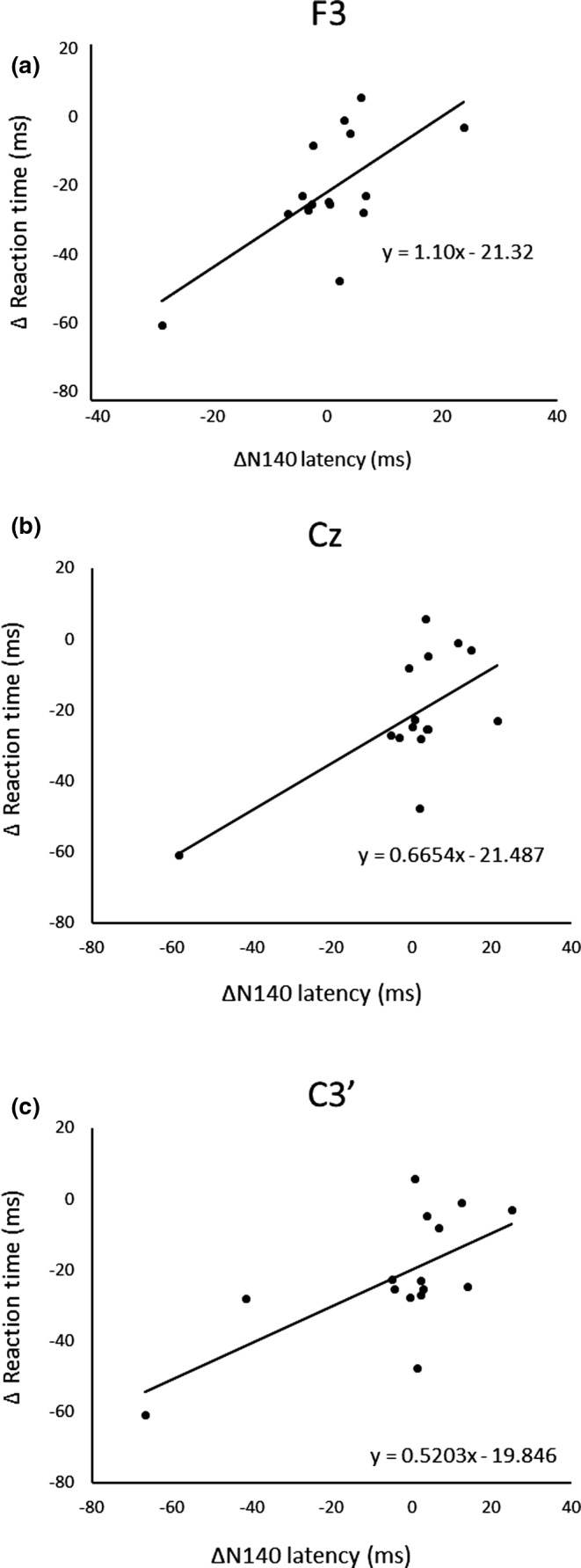
Correlations between ΔRT and ΔN140_lat_ at (a) F3, (b) Cz, and (c) C3’

**FIGURE 5:**
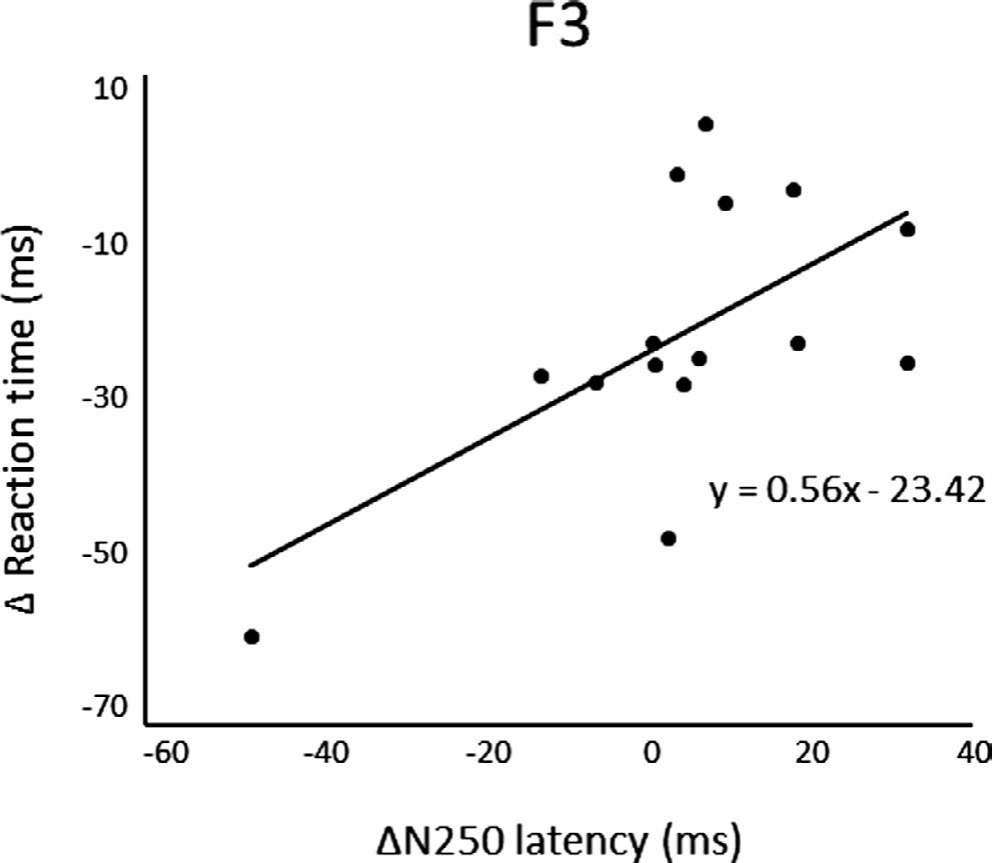
Correlations between ΔRT and ΔN250_lat_ at F3

**FIGURE 6:**
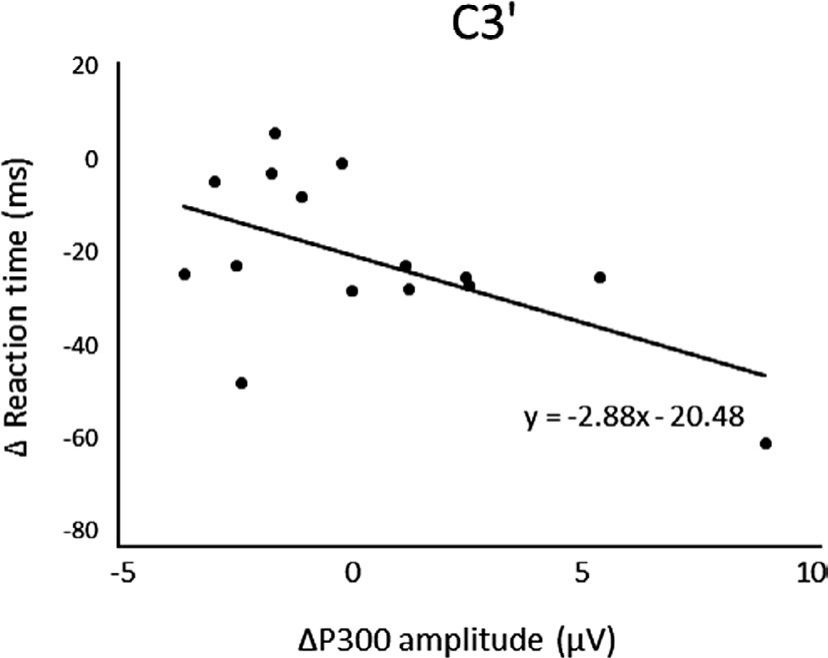
Correlations between ΔRT and ΔN300_amp_ at C3′

After three days of practice, 14 participants demonstrated a reduction in RT, and overall, there was a significant RT decrease from pre‐ to postpractice [*t*(14) = 4.71, *p* < .01] (Figure 1). Figure 2 shows the transition of RT from pre‐practice to postpractice, including the practice for 3 days. The average waveforms of more than 300 trials across six sets in pre‐ and postpractice for a typical participant are depicted in Figure 3. Table 1 summarizes the SEP amplitudes and the latencies at F3, Cz, and C3′ in pre‐ and postpractice and the change subtracted amplitude or latency of each SEP component in pre‐practice from the SEP component in postpractice. The amplitudes and latencies of all SEP components in all electrode sites showed no significant differences between pre‐ and postpractice. Correlation analyses revealed no significant relationship between the change in RT (ΔRT) and either the change in P100 amplitude (ΔP100_amp_) or P100 latency (ΔP100_lat_) at any electrode site. There was also no correlation between ΔRT and ΔN140_amp_ at any electrode site. However, Spearman's correlation coefficient was calculated between ΔRT and ΔN140_lat_ at F3, Cz, and C3′, and there was a significant positive correlation (F3, ρ = 0.52, *p* < .05; Cz, ρ = 0.56, *p* < .05; C3′, ρ = 0.60, *p* < .05) (Figure 4a–c). There was no significant correlation between ΔRT and ΔN250_amp_ at any electrode site, but Spearman's correlation coefficient was calculated between ΔRT and ΔN250_lat_ at F3; there was significantly and positively correlated (ρ = 0.59, *p* < .05) (Figure 5). Pearson's correlation coefficient was calculated between ΔRT and ΔP300_amp_ at C3′, there was a significant negative correlation (*r* = −.53, *p* < .05; Figure 6), but not between ΔRT and ΔP300_lat_ at any electrode site.

**TABLE 1:** Amplitude and latency of somatosensory evoked potential (SEP) components in pre‐ and postpractice for 3 days and the changes subtracted amplitude or latency of each SEP component in pre‐practice from the SEP component in postpractice

Electrode	Component	Amplitude (µV)			Latency (ms)		
		Pre	Post	Change	Pre	Post	Change
F3	P100	7.6 (3.7)	8.1 (3.8)	0.5 (3.1)	97.4 (15.5)	99.4 (14.2)	2.0 (7.6)
	N140	4.1 (3.2)	4.1 (3.2)	0.0 (0.8)	137.3 (19.4)	137.1 (16.2)	−0.1 (10.4)
	N250	6.1 (2.1)	6.7 (3.7)	0.6 (3.6)	242.7 (28.6)	246.1 (20.3)	3.4 (18.7)
	P300	6.0 (4.1)	6.3 (5.6)	0.3 (6.2)	334.0 (44.7)	309.7 (47.8)	−24.4 (43.1)
Cz	P100	9.7 (3.4)	9.7 (3.9)	0.0 (2.9)	86.1 (13.6)	91.1 (13.39	5.0 (11.1)
	N140	5.9 (3.1)	6.7 (2.4)	0.8 (2.7)	135.3 (18.4)	135.3 (10.5)	0.0 (17.0)
	N250	5.5 (3.3)	6.6 (4.6)	1.2 (4.5)	238.4 (36.7)	239.1 (28.7)	0.7 (20.6)
	P300	5.0 (3.2)	5.4 (2.2)	0.4 (3.1)	315.0 (50.7)	309.9 (46.3)	−5.1 (23.6)
C3'	P100	7.3 (3.1)	6.9 (4.6)	−0.4 (3.0)	88.0 (8.4)	88.4 (10.4)	0.4 (10.3)
	N140	6.0 (3.5)	6.9 (2.8)	0.9 (1.9)	138.4 (21.5)	135.3 (13.5)	−3.1 (21.8)
	N250	3.8 (4.0)	4.5 (3.7)	0.7 (2.6)	229.1 (20.6)	226.1 (21.8)	−3.0 (12.8)
	P300	5.3 (3.1)	5.6 (3.0)	0.3 (3.3)	281.7 (34.5)	282.0 (36.6)	0.2 (33.3)

Note

Data are expressed as means (standard deviations).

## 4 DISCUSSION

After 3 days of practice, significant differences were found in RT, but there were no significant differences in the amplitudes and latencies of SEP components, such as P100, N140, N250, and P300, between pre‐ and postpractice. Although one participant could not reduce RT, the RT of the motor response was shorter when the arousal or attention level was higher. The participants were asked to perform a very simple task, that is, just oppose their right thumb in response to electrical stimuli, and therefore, they may lose interest in the task and be distracted. In contrast, we analyzed the correlations in the change in RT and each SEP component between pre‐ and postpractice and found a significant positive correlation between ΔRT and ΔN140_lat_ at F3, Cz, and C3′, ΔRT and ΔN250_lat_ at F3, but there was a significant negative correlation between ΔRT and ΔP300_amp_ at C3′.

It has been reported in previous studies that the N140 amplitude increases during selective attention tasks requiring discrimination, such as the oddball task (Desmedt & Robertson, 1977; Michie, Bearpark, Crawform, & Glue, 1987). In addition, the N140 amplitude was enhanced by voluntary movements, but not passive movements (Nakata, Inui, Wasaka, Nishihara, & Kakigi, 2003). Moreover, the N140 amplitude was larger in a difficult stimulus discrimination task compared to a simple RT task (Kida, Nishihara, Wasaka, Sakajiri, & Tazoe, 2004). Thus, N140 appears to reflect the degree of attention to the stimulus required by the task. Yamashiro et al. (2013) also found shorter P100 and N140 latencies in baseball players compared to other athletes during index finger stimulation of the dominant hand. Baseball players require superior somatosensory processing in the cortical hand area for catching, batting, and throwing compared to athletes in sports like soccer. Repetitive grasping movements facilitate neuroplastic alterations in the cortical hand field, resulting in faster processing as reflected by reduced P100 and N140 peak latencies. Shorter N140 latencies have also been reported in recent studies in athletes compared to nonathletes (Bulut, Ozmerdivenli, & Bayer, 2003; Iwadate, Mori, Ashizuka, Takayose, & Ozawa, 2005). Therefore, long‐term training can alter SEPs by inducing neuroplastic alterations in specific cortical areas. In the current study, sensorimotor task practice for three days must have also altered task‐relevant sensorimotor networks as a shorter RT was associated with a reduced N140 peak latency. The generator of the N140 is still uncertain, but it has been reported in recent studies that the anterior cingulate cortex (ACC) is activated from 128 to 150 ms following somatosensory stimulation, suggesting a contribution to N140 (Gobbelé et al., 2008; Tanaka et al., 2008; Waberski, Gobbele, Darvas, Schmidt, & Buchner, 2002). Moreover, the ACC is considered to serve attentional, cognitive, and emotional functions (Tanaka et al., 2008), so it is possible that the reduced N140 latency and RT are related to faster processing by circuits involving the ACC. We speculate that more efficient pathways or shortcut circuits are formed by practice‐associated neuroplasticity, resulting in information reaching the ACC earlier than before practice.

It has been suggested in previous studies that the N250 may reflect conscious target detection (Jossiasen, Shagass, Roemer, Ercegovac, & Straumanis, 1982; Kekoni et al., 1996) as it is effectively elicited by rare target stimuli (Kujala et al., 1995). In addition, Kida et al. (2003) and Kida et al. (2003) found that the N250 latency was strongly correlated with the RT. The cerebral generators of the somatosensory N250 are still unknown, but it appears that reduced latency concomitant with shorter task RT following practice reflects more rapid decision‐making (Kida et al., 2003; Kida et al., 2003). However, the simple RT task used in the current study required only detection of the somatosensory stimulus, so we cannot assume that changes in the N250 component reflect higher‐order cognitive processes like decision‐making. Further studies with more difficult tasks are required to assess the significance of the N250 change.

In contrast to these other SEP waveforms, in which amplitude was unchanged by practice, the P300 amplitude increased in association with reduced RT. Enhancement of the P300 amplitude is associated with increases in the delay between a given target and the preceding target (Gonsalves, Barry, Rushby, & Polich, 2007). It has been reported in previous studies that the P300 amplitude is proportional to the attentional resources devoted to a given task (Kok, 1997, 2001). Therefore, the amplitude of P300 may provide an indication of the amount of attention required for task success. Kida et al. (2003) and Kida et al. (2003) suggested that larger P300 in fast‐RT trials reflects faster stimulus evaluation due to greater attentional resources. Athletes also demonstrated a significantly larger P300 amplitude compared to nonathletes (Iwadate et al., 2005). In summary, we suggest that higher‐level performance is associated with greater attention to the task. It is possible that improvements to somatosensory processing, decision‐making, and allocation of attentional resources lead to improvements of task performance. In contrast to our results, Iwadate et al. (2005) found that athletes demonstrated a significantly shorter P300 latency compared to nonathletes and suggested that this latency reflects the stimulus evaluation time (Kutas, McCarthy, & Donchin, 1977). We suggest that the absence of an association between RT and ΔP300_lat_ in our study reflects low task demands. The simple RT task is easier than the oddball task used by Iwadate et al. (2005), so allocation of attention was less important for performance. Alternatively, the subjects in the aforementioned study received long‐term training, so it is possible that three days of practice may be insufficient to alter the P300 latency.

There was no significant correlation between RT and P100 amplitude or latency. It has been suggested in previous studies that P100 and N140 reflect passive attention and that P100 is generated mainly by the secondary somatosensory cortex (SII) (Tanaka et al., 2008). It has been reported in magnetoencephalography (MEG) studies that SII is activated 70–140 ms after somatosensory stimulation and is involved in higher‐order processing for somatosensory perception (Mima, Nagamine, Nakamura, & Shibasaki, 1998) and integration with motor actions (Hari & Forss, 1999). The generators of P100 and N140 may not coincide and so could be activated independently, resulting in distinct associations with RT.

In conclusion, our results show N140 and N250 latency, which reflect the attention to the stimulus, and P300 amplitude, which is proportional to the attentional resources devoted to a given task, were altered by repetitive practice. Previous studies have reported that attentional neuromodulation occurred in athletes who have trained for a long term (Bulut et al., 2003; Iwadate et al., 2005); however, our results suggested that short‐term training such as that for 3 days can also induce neuroplasticity related to attention.

## CONFLICT OF INTEREST

None.

## AUTHOR CONTRIBUTIONS

AM and SK conceived and designed research, and revised the paper; AM, IK, SH, and SK collected data and conducted research; AM, IK, ST, and SK analyzed and interpreted data; AM wrote the initial paper; SK had primary responsibility for final content. All authors read and approved the final manuscript.

## INFORMED CONSENT

Informed consent was obtained from all individual participants included in the study.

## Data Availability

The datasets generated and analyzed during the present study are available from the corresponding author on reasonable request.

## References

[brb31624-bib-0001] Bulut, S. , Ozmerdivenli, R. , & Bayer, H. (2003). Effects of exercise on somatosensory‐evoked potentials. International Journal of Neuroscience, 113, 315–322. 10.1080/00207450390162119 12803136

[brb31624-bib-0002] Desmedt, J. E. , & Robertson, D. (1977). Differential enhancement of early and late component of the cerebral somatosensory evoked potentials during forced‐paced cognitive tasks in man. Journal of Physiology, 271, 61–82. 10.1113/jphysiol.1977.sp012025 PMC1353632 926022

[brb31624-bib-0003] Donchin, E. , & Coles, M. G. H. (1988). Is the P300 component a manifestation of context updating? Behavioral and Brain Sciences, 100, 357–374. 10.1017/S0140525X00058027

[brb31624-bib-0004] Gobbelé, R. , Lamberty, K. , Stephan, K. E. , Stegelmeyer, U. , Buchner, H. , Marshall, J. C. , … Waberski, T. D. (2008). Temporal activation patterns of lateralized cognitive and task control processes in the human brain. Brain Research, 1205, 81–90. 18353286 10.1016/j.brainres.2008.02.031

[brb31624-bib-0005] Gonsalves, C. J. , Barry, R. J. , Rushby, J. A. , & Polich, J. (2007). Target‐to‐target interval, intensity, and P300 from an auditory single‐stimulus task. Psychophysiology, 44, 245–250. 10.1111/j.1469-8986.2007.00495.x 17343708

[brb31624-bib-0006] Hari, R. , & Forss, N. (1999). Magnetoencephalography in the study of human somatosensory cortical processing. Philosophical Transactions of the Royal Society of London. Series B, Biological Sciences, 29, 1145–1154. 10.1098/rstb.1999.0470 PMC1692629 10466142

[brb31624-bib-0007] Hiraoka, K. , Kori, C. , & Sato, K. (2017). The somatosensory‐evoked potential in reaction time is gated and elicited earlier when the motor response to a somatosensory cue is faster. NeuroReport, 28, 451–456. 10.1097/WNR.0000000000000784 28430708

[brb31624-bib-0008] Iwadate, M. , Mori, A. , Ashizuka, T. , Takayose, M. , & Ozawa, T. (2005). Long‐term physical exercise and somatosensory event‐related potentials. Experimental Brain Research, 160, 528–532. 10.1007/s00221-004-2125-5 15586274

[brb31624-bib-0009] Jossiasen, R. C. , Shagass, C. , Roemer, R. A. , Ercegovac, D. V. , & Straumanis, J. J. (1982). Somatosensory evoked potential changes with a selective attention task. Psychophysiology, 19, 146–159. 10.1111/j.1469-8986.1982.tb02536.x 7071293

[brb31624-bib-0010] Kekoni, J. , Hamalainen, H. , McCloud, V. , Reinekainen, K. , & Naatanen, R. (1996). Is the somatosensory N250 related to deviance discrimination or conscious target detection? Electroencephalography and Clinical Neurophysiology, 100, 115–125. 10.1016/0013-4694(95)00231-6 8617150

[brb31624-bib-0011] Kida, T. , Nishihara, Y. , Hatta, A. , & Wasaka, T. (2003). Somatosensory N250 and P300 during discrimination tasks. International Journal of Psychophysiology, 48, 275–283. 10.1016/S0167-8760(03)00021-7 12798987

[brb31624-bib-0012] Kida, T. , Nishihara, Y. , Hatta, A. , Wasaka, T. , Nakata, H. , Sakamoto, M. , & Nakajima, T. (2003). Changes in the somatosensory N250 and P300 by the variation of reaction time. European Journal of Applied Physiology, 89, 326–330. 10.1007/s00421-003-0801-y 12736841

[brb31624-bib-0013] Kida, T. , Nishihara, Y. , Wasaka, T. , Sakajiri, Y. , & Tazoe, T. (2004). Differential modulation of the short‐ and long‐latency somatosensory evoked potentials in a forewarned reaction time task. Clinical Neurophysiology, 115, 2223–2230. 10.1016/j.clinph.2004.04.017 15351362

[brb31624-bib-0014] Kok, A. (1997). Event‐related‐potential (ERP) reflections of mental resources: A review and synthesis. Biological Psychology, 45, 19–56. 9083643 10.1016/s0301-0511(96)05221-0

[brb31624-bib-0015] Kok, A. (2001). On the utility of P3 amplitude as a measure of processing capacity. Psychophysiology, 38, 557–577. 10.1017/S0048577201990559 11352145

[brb31624-bib-0016] Kujala, T. , Alho, K. , Kekoni, J. , Hamalainen, H. , Reinikainen, K. , Salonen, O. , … Naatanen, R. (1995). Auditory and somatosensory event‐related brain potentials in early blind humans. Experimental Brain Research, 104, 519–526. 10.1007/BF00231986 7589303

[brb31624-bib-0017] Kutas, M. , McCarthy, G. , & Donchin, E. (1977). Augmenting mental chronometry: The P300 as a measure of stimulus evaluation time. Science, 197, 792–795. 10.1126/science.887923 887923

[brb31624-bib-0018] Michie, P. T. (1984). Selective Attention Effects on Somatosensory Event‐Related Potentials. Annals of the New York Academy of Sciences., 425(1 Brain and Inf), 250–255. 6588840 10.1111/j.1749-6632.1984.tb23542.x

[brb31624-bib-0019] Michie, P. T. , Bearpark, H. M. , Crawform, J. M. , & Glue, L. C. (1987). The effects of spatial selective attention on the somatosensory event‐related potential. Psychophysiology, 4, 449–463. 10.1111/j.1469-8986.1987.tb00316.x 3615757

[brb31624-bib-0020] Mima, T. , Nagamine, T. , Nakamura, K. , & Shibasaki, H. (1998). Attention modulates both primary and second somatosensory cortical activities in humans: A magnetoencephalographic study. Journal of Neurophysiology, 80, 2215–2221. 10.1152/jn.1998.80.4.2215 9772274

[brb31624-bib-0021] Nakata, H. , Inui, K. , Wasaka, T. , Nishihara, Y. , & Kakigi, R. (2003). Mechanisms of differences in gating effects on short‐and long‐latency somatosensory evoked potentials relating to movement. Brain Topography, 15, 211–222. 12866825 10.1023/a:1023908707851

[brb31624-bib-0022] Sugawara, K. , Onishi, H. , Yamashiro, K. , Soma, T. , Oyama, M. , Kirimoto, H. , … Kameyama, S. (2013). Repeated practice of a Go/NoGo visuomotor task induces neuroplastic change in the human posterior parietal cortex: An MEG study. Experimental Brain Research, 226, 495–502. 10.1007/s00221-013-3461-0 23455731

[brb31624-bib-0023] Tanaka, E. , Inui, K. , Kida, T. , Miyazaki, T. , Takeshima, Y. , & Kakigi, R. (2008). A transition from unimodal to multimodal activations in four sensory modalities in humans: an electrophysiological study. BMC Neuroscience., 9(1). 10.1186/1471-2202-9-116 PMC2607283 19061523

[brb31624-bib-0024] Waberski, T. D. , Gobbele, R. , Darvas, F. , Schmidt, Z. , & Buchner, H. (2002). Spatiotemporal imaging of electrical activity related to attention to somatosensory stimulation. NeuroImage, 17, 1347–1357. 10.1006/nimg.2002.1222 12414274

[brb31624-bib-0025] Yamashiro, K. , Sato, D. , Onishi, H. , Yoshida, T. , Horiuchi, Y. , Nakazawa, S. , & Maruyama, A. (2013). Skill‐specific changes in somatosensory‐evoked potentials and reaction times in baseball players. Experimental Brain Research, 225, 197–203. 10.1007/s00221-012-3361-8 23224701

[brb31624-bib-0026] Yotani, K. , Tamaki, H. , Yuki, A. , Kirimoto, H. , Kitada, K. , Ogita, F. , & Takekura, H. (2011). Response training shortens visuomotor related time in athletes. International Journal of Sports Medicine, 32, 586–590. 10.1055/s-0031-1275299 21563022

